# Perceived satisfaction with HIV care and its association with adherence to antiretroviral therapy and viral suppression in the African Cohort Study

**DOI:** 10.1186/s12981-021-00414-3

**Published:** 2021-11-25

**Authors:** Nancy Somi, Nicole Dear, Domonique Reed, Ajay Parikh, Anange Lwilla, Emmanuel Bahemana, Samoel Khamadi, Michael Iroezindu, Hannah Kibuuka, Jonah Maswai, Trevor A. Crowell, John Owuoth, Lucas Maganga, Christina Polyak, Julie Ake, Allahna Esber, the AFRICOS Study Group

**Affiliations:** 1 HJF Medical Research International, Mbeya, Tanzania; 2 U.S. Military HIV Research Program, Walter Reed Army Institute of Research, Silver Spring, MD USA; 3 Henry M. Jackson Foundation for the Advancement of Military Medicine Inc., 6720a Rockledge Drive, Suite 400, Bethesda, MD 20817 USA; 4 HJF Medical Research International, Abuja, Nigeria; 5 Makerere University Walter Reed Project, Kampala, Uganda; 6 HJF Medical Research International, Kericho, Kenya; 7 U.S. Army Medical Research Directorate – Africa, Kisumu, Kenya; 8 HJF Medical Research International, Kisumu, Kenya; 9 National Institute for Medical Research – Mbeya Medical Research Center, Mbeya, Tanzania

**Keywords:** HIV/AIDS, Quality of care, Viral load

## Abstract

**Background:**

Increased availability of HIV care over the past decade has dramatically reduced morbidity and mortality among people living with HIV (PLWH) in sub-Saharan Africa. However, perceived and experienced barriers to care, including dissatisfaction with services, may impact adherence and viral suppression. We examined the associations between satisfaction with HIV care and antiretroviral therapy (ART) adherence and viral load suppression.

**Methods:**

The African Cohort Study (AFRICOS) is a prospective observational study conducted at PEPFAR-supported clinics in four African countries. At enrollment and twice-yearly study visits, participants received a clinical assessment and a socio-behavioral questionnaire was administered. Participants were classified as dissatisfied with care if they reported dissatisfaction with any of the following: waiting time, health care worker skills, health care worker attitudes, quality of clinic building, or overall quality of care received. Robust Poisson regression was used to estimate prevalence ratios and 95% confidence intervals (CIs) for associations between satisfaction with care and ART adherence and between satisfaction with care and viral suppression (viral load < 1000 copies/mL).

**Results:**

As of 1 March 2020, 2928 PLWH were enrolled and 2311 had a year of follow-up visits. At the first annual follow-up visit, 2309 participants responded to questions regarding satisfaction with quality of care, and 2069 (89.6%) reported satisfaction with care. Dissatisfaction with waiting time was reported by 177 (7.6%), building quality by 59 (2.6%), overall quality of care by 18 (0.8%), health care worker attitudes by 16 (0.7%), and health care worker skills by 15 (0.7%). After adjusting for age and site, there was no significant difference in viral suppression between those who were satisfied with care and those who were dissatisfied (aPR: 1.03, 95% CI 0.97–1.09). Satisfaction with HIV care was moderately associated with ART adherence among AFRICOS participants (aPR: 1.09; 95% CI 1.00–1.16).

**Conclusions:**

While patient satisfaction in AFRICOS was high and the association between perceived quality of care and adherence to ART was marginal, we did identify potential target areas for HIV care improvement, including reducing clinic waiting times.

**Supplementary Information:**

The online version contains supplementary material available at 10.1186/s12981-021-00414-3.

## Background

The widespread rollout and use of antiretroviral therapy (ART) in sub-Saharan Africa in the last two decades remains a monumental public health achievement [1]. Effective ART has made an impact on the clinical course of HIV infection, and has reduced disease progression, incidence of opportunistic infections, and mortality in sub-Saharan Africa [2]. While use of ART has led to a dramatic decrease in morbidity and mortality, nonadherence remains a major challenge [3].

The third component of the UNAIDS 95-95-95 target, viral load suppression for 95% of people living with HIV (PLWH) on ART [4], is directly linked to ART adherence. Nonadherence to ART and uncontrolled viremia remain a major cause of HIV-related morbidity and mortality [5], and have the potential to impede progress towards the third 95. Several studies have identified factors associated with nonadherence, including lack of self-motivation or diagnosis acceptance, socio-economic status, forgetfulness, lack of social support and lack of trust in a care provider [6–9]. A cohort study across six African countries found additional barriers to adherence as self-reported by PLWH, including sickness or adverse events, pharmacy stock outs, stigma or depression, pill burden and regimen complexity [10]. Quality and holistic care for PLWH is key to sustaining high levels of adherence and long-term viral suppression [11].

As well-established HIV care and treatment programs transition from rapid ART scale-up [12], it is increasingly recognized that quality improvements can help close gaps along the care cascade and improve clinical outcomes [13, 14]. A systematic review by Hargreaves et al., found that quality improvement initiatives in HIV programs in low and middle income countries contributed most to ART uptake, ART adherence and viral suppression, within the context of national policy and program changes [13]. Importantly, patient satisfaction and perceived quality of care, including in regards to health personnel proficiency, healthcare delivery, sufficiency of resources and services, accessibility and cost of care, are important to the overall wellbeing of PLWH [15, 16]. Variable evidence about which aspects of quality interventions lead to the greatest improvements of care suggest that better standardization and further research is needed [13].

Given the suggested relationship between satisfaction with HIV care, adherence, and viral suppression, improving the quality of HIV care in sub-Saharan Africa may help shrink existing gaps in achieving the UNAIDS 95-95-95 targets [4]. We assessed the association between satisfaction with HIV care and adherence to ART and viral load suppression in four African countries.

## Methods

### Study design and participants

As previously described, the African Cohort Study (AFRICOS) is an ongoing prospective cohort study enrolling PLWH and HIV uninfected adult participants at 12 President’s Emergency Plan for AIDS Relief (PEPFAR)-supported HIV care and treatment clinics across five study sites in four countries: Tanzania, Kenya (Kisumu and South Rift Valley) Uganda and Nigeria [17]. PLWH were selected randomly from current client lists, with a small subset recruited from prior HIV research studies. All non-pregnant, non-incarcerated adults age 18 years and older consenting to data and specimen collection were eligible for inclusion.

The study was approved by the institutional review boards of the Walter Reed Army Institute of Research (#1897), Makerere University School of Public Health (#173), Uganda National Committee of Science and Technology (HS-1175), Kenya Medical Research Institute Science and Ethics Review Unit (SSC# 2396, 2371), Tenwek Institutional Ethics Review Committee (SSC# 2371), Tanzania National Institute of Medical Research (NIMR/HQ/R.8a/Vol.1X/1060), Mbeya Medical and Research Ethics Committee (NIMR/HQ/R.8a/Vol.1X/1060), and Ministry of Defense Health Research and Ethics Committee (#3726112019). All participants gave written informed consent.

### Data collection and measures

At enrollment and twice-yearly study visits, participants received a clinical assessment and a structured socio-behavioral questionnaire was administered by trained clinic staff through face to face interviews. The subject questionnaire collected data on demographics, including age, sex, employment status, educational attainment, food security (defined as having enough food to eat in the past 12 months), and healthcare accessibility (defined as distance from facility).

Participants were asked a series of questions regarding their satisfaction with services received at the ART clinic. Topics included satisfaction with the following: waiting time, health care worker skills, health care worker attitudes, quality of clinic building, and overall quality of care received (Additional file 1: Fig. S1). For these analyses, perception on quality of care was measured as either satisfied or dissatisfied. Participants were classified as dissatisfied with care if they reported dissatisfaction with any of the items.

ART adherence was based on the self-reported number of doses missed in the past 30 days. Participants were classified as nonadherent if that had missed one or more doses in the past 30 days. Viral suppression was defined as a viral load < 1000 copies/mL.

Data were captured on paper case report forms then entered and verified in the ClinPlus platform (Anju Software, Tempe, AZ).

### Statistical analyses

Chi-squared and Wilcoxon rank sum tests were used to describe significant differences in participant satisfaction by select demographic and HIV-related characteristics at the first annual visit after enrollment. Generalized linear models with a Poisson distribution and robust standard errors were used to estimate unadjusted and adjusted prevalence ratios (aPRs) and 95% confidence intervals (95% CIs) for associations between satisfaction with care and ART adherence and between satisfaction with care and viral suppression. Confounding was assessed using a 10% change in coefficients. Each model was restricted to observations with non-missing data for all variables included in the unadjusted and adjusted models.

All analyses were performed in SAS version 9.4 (SAS Institute, Cary, North Carolina) and Stata version 16.0 (StataCorp, College Station, Texas) software.

## Results

### Study population characteristics

As of March 1, 2020, 2928 PLWH were enrolled in the African Cohort Study with 2311 having at least 1 year of follow-up visits. Of these, 2309 participants responded to questions regarding satisfaction with quality of care at the HIV care and treatment clinics at the first annual follow up-visit. The median age was 40.3 (interquartile range [IQR]: 33.5–47.7) years and 1351 (58.5%) were female (Table 1). Most participants had a primary level education or less (n = 1390, 60.2%), 1378 (59.7%) were unemployed, and 1602 (69.4%) had enough food to eat in the past 12 months. Participants lived a median of 8 km from their HIV care facility (IQR: 4.5–16 km) and distance did not differ significantly by satisfaction with care. Overall, the median time since HIV diagnosis was 4.1 (IQR: 1.4–7.5) years and median time on ART was 2.5 (IQR: 1.1–6.1) years.

**Table 1:** Characteristics of PLWH by perceived satisfaction with care at first annual visit after enrollment

	All participants (n = 2309)	Dissatisfied (n = 240)	Satisfied (n = 2069)	P-value
Study site				***< 0.001***
Kayunga, Uganda	421 (18.2%)	53 (22.1%)	368 (17.8%)	
South Rift Valley, Kenya	825 (35.7%)	41 (17.1%)	784 (37.9%)	
Kisumu West, Kenya	460 (19.9%)	28 (11.7%)	432 (20.9%)	
Mbeya, Tanzania	411 (17.8%)	68 (28.3%)	343 (16.6%)	
Abuja & Lagos Nigeria	192 (8.3%)	50 (20.8%)	142 (6.9%)	
Age (years)				*0.69*
18–29	323 (14.0%)	39 (16.3%)	284 (13.7%)	
30–39	802 (34.7%)	78 (32.5%)	724 (35.0%)	
40–49	720 (31.2%)	73 (30.4%)	647 (31.3%)	
50+	464 (20.1%)	50 (20.8%)	414 (20.0%)	
Sex				*0.54*
Male	958 (41.5%)	104 (43.3%)	854 (41.3%)	
Female	1351 (58.5%)	136 (56.7%)	1215 (58.7%)	
Currently employed				***< 0.001***
No	1378 (59.7%)	110 (45.8%)	1268 (61.3%)	
Yes	931 (40.3%)	130 (54.2%)	801 (38.7%)	
Education				***< 0.001***
Primary or less	1390 (60.2%)	116 (48.3%)	1274 (61.6%)	
Secondary or above	919 (39.8%)	124 (51.7%)	795 (38.4%)	
Enough food to eat in past 12 months				***0.03***
No	706 (30.6%)	58 (24.2%)	648 (31.3%)	
Yes	1602 (69.4%)	181 (75.4%)	1421 (68.7%)	
Missing	1 (<1%)	1 (0.4%)	0 (0.0%)	
Distance from facility (km), median (IQR)	8 (4.5-16.0)	9 (4.5-20.0)	8 (4.5-16.0)	*0.78*
Time since HIV diagnosis				*0.09*
< 1 year	27 (1.2%)	3 (1.3%)	24 (1.2%)	
1 to 4 years	1250 (54.1%)	137 (57.1%)	1113 (53.8%)	
5 to 9 years	789 (34.2%)	68 (28.3%)	721 (34.8%)	
10 or more years	218 (9.4%)	31 (12.9%)	187 (9.0%)	
Missing	25 (1.1%)	1 (0.4%)	24 (1.2%)	
Duration on ART				***0.03***
ART naïve	131 (5.7%)	16 (6.7%)	115 (5.6%)	
< 6 months	79 (3.4%)	16 (6.7%)	63 (3.0%)	
6 months to < 2 years	889 (38.5%)	82 (34.2%)	807 (39.0%)	
2 years to < 4 years	341 (14.8%)	32 (13.3%)	309 (14.9%)	
≥ 4 years	854 (37.0%)	92 (38.3%)	762 (36.8%)	
Missing	15 (0.6%)	2 (0.8%)	13 (0.6%)	

All data are presented as n (column percentage). P-values were calculated using Pearson’s chi-squared tests or Wilcoxon rank sum tests for continuous variables. Bold indicates significance at p < 0.05

### AFRICOS participants’ perception on quality of HIV care

Overall, 2069 (89.6%) reported being satisfied with their HIV care while 240 (10.4%) reported dissatisfaction with at least one of the indicators at their first annual follow-up visit (Table 1). Satisfaction with care did not vary by sex, with 104 (10.9%) males and 136 (10.1%) females reporting dissatisfaction with HIV care (p = 0.54). As compared to unemployed participants, a greater proportion of participants who were employed reported being dissatisfied with care (54.2% vs. 45.8%, p < 0.001). A greater proportion of those with a secondary level education or above were dissatisfied as compared to those with primary level education or less (51.7% vs. 48.3%, p < 0.001). As compared to those who did not have enough food to eat in the past 12 months, a greater proportion of those who had enough food to eat in the past 12 months reported dissatisfaction with care (75.4% vs. 24.2%, p = 0.03). Satisfaction with care also varied significantly by study site (p < 0.001). The primary driver of dissatisfaction was waiting time (n = 177, 7.7%), followed by building quality (n = 59, 2.6%), overall quality of care (n = 18, 0.8%), health care workers attitudes (n = 16, 0.7%), and health care worker skills (n = 15, 0.7%; Table 2). Examining each individual component of dissatisfaction with care, study site differed significantly among those dissatisfied with waiting time, building quality, health care workers attitudes, and health care worker skills (Additional file 1: Table S1).

**Table 2:** Proportion of participants dissatisfied with individual components of HIV care services

	n = 2309	%
Waiting time	177	7.66
Building quality	59	2.55
Overall quality of care	18	0.78
Healthcare workers attitudes	16	0.69
Healthcare worker skills	15	0.65

### Quality of care, viral suppression and ART adherence

Satisfaction with care was associated with ART adherence in the unadjusted models (PR: 1.15; 95% CI 1.06–1.24; Table 3). Though slightly attenuated, the association remained moderately significant after adjustment for study site and age (aPR: 1.08; 95% CI 1.00–1.16).

Among participants included in these analyses, 87.5% were virally suppressed at their first annual follow-up visit. Satisfaction with care was not associated with viral suppression in the unadjusted models (PR: 1.04, 95% CI 0.98–1.11; Table 4), and remained nonsignificant after adjustment for study site and age (aPR: 1.03, 95% CI 0.97–1.09).

**Table 3:** Unadjusted and adjusted PR for association between satisfaction with care and ART adherence at first annual visit after enrollment

	PR (95% CI)	aPR (95% CI)
Satisfaction with care		
Needs improvement	Ref	–
Doesn’t need improvement	**1.15 (1.06–1.24)**	**1.08 (1.00–1.16)**
Study site		
Kayunga, Uganda	Ref	–
South Rift Valley, Kenya	**1.17 (1.11–1.23)**	**1.16 (1.09–1.22)**
Kisumu West, Kenya	**1.09 (1.03–1.16)**	**1.09 (1.03–1.16)**
Mbeya, Tanzania	1.03 (0.96–1.10)	1.03 (0.97–1.11)
Abuja & Lagos Nigeria	**0.80 (0.72-0.91)**	**0.81 (0.72–0.91)**
Age (years)		
18–29	Ref	–
30–39	1.04 (0.98-1.11)	1.04 (0.97-1.11)
40–49	**1.10 (1.03-1.17)**	**1.09 (1.03–1.16)**
50+	**1.11 (1.04-1.18)**	**1.08 (1.02–1.15)**
Sex		
Male	Ref	
Female	1.01 (0.97–1.04)	
Currently employed		
No	Ref	
Yes	**0.90 (0.87–0.94)**	
Education		
Primary or less	Ref	
Secondary or above	0.98 (0.95–1.02)	
Enough food to eat in past 12 months		
No	Ref	
Yes	0.98 (0.94–1.01)	
Distance from facility (km)	1.00 (1.00–1.00)	
Time since HIV diagnosis		
< 1 year	Ref	
1 to 4 years	1.06 (0.86–1.31)	
5 to 9 years	1.11 (0.90–1.37)	
10 or more years	1.12 (0.91–1.38)	
Duration on ART		
ART naïve	Ref	
< 6 months	**0.91 (0.85-0.98)**	
6 months to < 2 years	**0.83 (0.81–0.86)**	
2 years to < 4 years	**0.84 (0.80–0.88)**	
≥ 4 years	**0.89 (0.87–0.91)**	

Robust Poisson regression was used to estimate prevalence ratios and 95% confidence intervals (CIs) for associations between satisfaction with care and ART adherence. Bold indicates significance at p < 0.05

**Table 4:** Unadjusted and adjusted PR for association between satisfaction with care and viral suppression at first annual visit after enrollment

	PR (95% CI)	aPR (95% CI)
Satisfaction with care		
Needs improvement	Ref	–
Doesn’t need improvement	1.04 (0.98-1.11)	1.03 (0.97–1.09)
Study site		
Kayunga, Uganda	Ref	–
South Rift Valley, Kenya	1.05 (1.00–1.10)	1.04 (0.99–1.09)
Kisumu West, Kenya	**1.07 (1.01-1.12)**	**1.06 (1.01-1.12)**
Mbeya, Tanzania	0.96 (0.91–1.02)	0.96 (0.91–1.02)
Abuja & Lagos Nigeria	1.02 (0.95–1.09)	1.01 (0.95–1.09)
Age (years)		
18–29	Ref	-
30–39	1.04 (0.98–1.10)	1.03 (0.97–1.10)
40–49	**1.11 (1.05–1.17)**	**1.10 (1.04–1.17)**
50+	**1.10 (1.03–1.17)**	**1.09 (1.03–1.16)**
Sex		
Male	Ref	
Female	0.98 (0.95–1.01)	
Currently employed		
No	Ref	
Yes	1.00 (0.97–1.03)	
Education		
Primary or less	Ref	
Secondary or above	1.00 (0.96–1.03)	
Enough food to eat in past 12 months		
No	Ref	
Yes	0.99 (0.95–1.02)	
Distance from facility (km)	1.00 (1.00–1.00)	
Time since HIV diagnosis		
< 1 year	Ref	
1 to 4 years	1.16 (0.92–1.47)	
5 to 9 years	1.24 (0.98–1.57)	
10 or more years	1.23 (0.97–1.56)	
Duration on ART		
ART naïve	Ref	
< 6 months	**4.40 (3.13–6.18)**	
6 months to < 2 years	**4.28 (3.06–5.99)**	
2 years to < 4 years	**4.36 (3.11–6.10)**	
≥ 4 years	**4.27 (3.05–5.98)**	

Robust Poisson regression was used to estimate prevalence ratios and 95% confidence intervals (CIs) for associations between satisfaction with care and viral suppression (viral load <1000 copies/mL). Bold indicates significance at p < 0.05

## Discussion

High quality HIV care in ART clinics is just as important as diagnosing, treating, preventing and controlling the disease in African countries as advocated by UNAIDS in 2014. Although African governments, assisted by donors and funders, are working hard to end the AIDS epidemic by 2030, little has been said about the quality of care provided by ministries of health [4]. Consistent with findings from similar studies, we found that the majority of AFRICOS study participants were satisfied with the quality of care they received [15]. A comparable study done in South Africa among 20 PLWH revealed similarly low levels of dissatisfaction [2]. A cross-sectional survey conducted in Nigeria assessing overall quality of care, staff attitude, confidentiality, distance to and time spent at facility indicated that PLWH were highly satisfied with overall quality of care and those who were dissatisfied with confidentiality and staff attitude had lower odds of satisfaction with overall quality of care [18].

Among participants dissatisfied with care, waiting time was given as the top reason. Waiting time was also reported by ART users at some ART facilities in Botswana, Ghana, Tanzania and Uganda as one of the main obstacles to optimal adherence [3, 19–21]. A study conducted among 408 PLWH in Nigeria indicates that participants are satisfied with care in general but not waiting time (73%) [22]. Another study conducted among PLWH who attended HIV private clinics in Dar-es- Salaam, Tanzania found that 19% wanted decreased waiting times despite waiting for less than 1 h [23]. This can be explained by the fact that most private clinics have shorter waiting times and are quick at responding to user needs compared to public clinics [24, 25]. To rectify long waiting times, studies suggest employing more staff members and scheduling patients at different times of the day [26]. Entertainment will also enable patients to stay engaged and wait actively; some suggested forms of entertainment are television, music and providing informational health reading materials [27, 28]. Reducing clinic waiting time or providing engaging waiting room activities could have downstream effects on ART adherence and overall health outcomes for PLWH.

Another component of care that participants reported dissatisfaction with was healthcare worker skills and attitudes. A similar study done in Singapore confirmed patients filed complaints which indicated dissatisfaction with doctors’ attitude/conduct (28.8%) and professional skills (17.8%) [20]. In this study, most participants reported being satisfied with care, but several had negative experiences regarding health care providers’ skills or attitudes, providing a specific target for improvement during healthcare worker training.

Study findings indicated no association between perceived quality of care and viral load suppression among AFRICOS participants. In contrast, a cross-sectional study conducted in Houston, Texas at an ART primary care clinic found that patients who were satisfied with care had higher odds of viral suppression compared to those who were not [29]. Perceived care may also indirectly affect viral suppression in situations where patients do not trust their care givers or were not satisfied with care, consequently missing care visits. Longer time spent without seeing physicians resulted in reduced viral suppression [29, 30].

Satisfaction with care was moderately associated with ART adherence in AFRICOS. This finding aligns with other settings where satisfaction with care influenced adherence. In Manaus, Brazil participants who rated the quality of care highly were approximately two times more likely to adhere to care than those who did not. In this study predictors of satisfaction with care included convenient location of the clinic, waiting time, and respectful treatment from the nurses [31]. These results are also comparable to the cross sectional study conducted in Houston, Texas where participants with high scores of satisfaction were more likely to adhere to care (p < 0.0001) [29]. Conversely a study conducted in the midwestern United States suggests that the majority of PLWH come to clinics based on their own motivation and consideration for their health rather than quality of care and influence from health care workers, thus it is not surprising we also did not detect a strong signal [7].

Limitations of this study include that the data around satisfaction with care were ascertained through face-to-face interviews with care providers, thus social desirability bias may have influenced how AFRICOS participants responded to these questions. Furthermore, participants enrolled in a longitudinal cohort study may receive enhanced attention and support and may not be representative of the general clinic populations in these settings. The existing survey only assessed summary measures of satisfaction and thus we were not able to delve into detailed aspects such as specific services provided by the health care worker. Future research should focus on other aspects of HIV care as well as explore country level differences that may impact on quality of HIV care and clinical outcomes differentially.

## Conclusions

Satisfaction with HIV care was reported by a majority of study participants at first annual follow-up visit. While there was no significant association between satisfaction and viral suppression and only a marginal association between satisfaction and ART adherence, reductions in clinic waiting time as well as improvements to healthcare worker training and proficiency may provide additional benefit to PLWH and HIV care programs overall. Rigorous and routine program evaluations are needed to ensure quality improvement initiatives translate into positive patient level and programmatic outcomes.

## Supplementary Information

**Additional file 1: Figure 1.** Participants were asked a series of questions regarding their satisfaction with services received at the ART clinic. **Table 1.** Characteristics of PLWH by dissatisfaction with individual components of care at first annual visit after enrollment.

## Data Availability

The Henry M. Jackson Foundation for the Advancement of Military Medicine (HJF) and the Water Reed Army Institute of Research (WRAIR) are committed to safeguarding the privacy of research participants. Distribution of data will require compliance with all applicable regulatory and ethical processes, including establishment and approval of an appropriate data-sharing agreement. To request a minimal data set, please contact the data coordinating and analysis center (DCAC) at PubRequest@hivresearch.org and indicate the RV329 study along with the name of the manuscript.
