# Supplementary material for: Perceived satisfaction with HIV care and its association with adherence to antiretroviral therapy and viral suppression in the African Cohort Study
Source: AIDS Res Ther. 2021 Nov 25;18:89. doi: 10.1186/s12981-021-00414-3 (PMC8614053; doi:10.1186/s12981-021-00414-3)
Supplement: Supplementary file 1 — Additional file 1: Figure 1. Participants were asked a series of questions regarding their satisfaction with services received at the ART clinic. Table 1. Characteristics of PLWH by dissatisfaction with individual components of care at first annual visit after enrollment. [file 12981_2021_414_MOESM1_ESM.docx]

Additional file

Figure S1. Participants were asked a series of questions regarding their satisfaction with services received at the ART clinic.


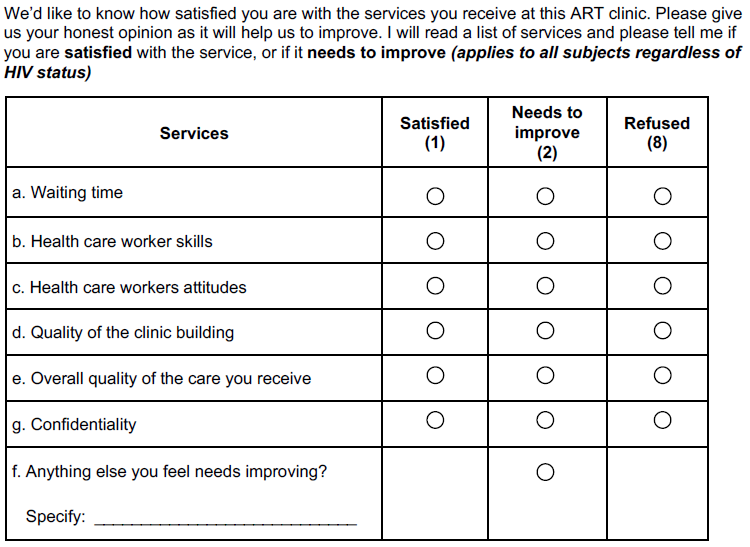


Table S1. Characteristics of PLWH by dissatisfaction with individual components of care at first annual visit after enrollment

|  | Dissatisfied with waiting time  (n=177) | Dissatisfied with building quality  (n=59) | Dissatisfied with overall quality of care  (n=18) | Dissatisfied with healthcare workers attitudes  (n=16) | Dissatisfied with  healthcare worker  skills  (n=15) |
| --- | --- | --- | --- | --- | --- |
| Study site |  |  |  |  |  |
| Kayunga, Uganda | **36 (20.3%)** | **24 (40.7%)** | 7 (38.9%) | **5 (31.3%)** | **0 (0.0%)** |
| South Rift Valley, Kenya | **29 (16.4%)** | **8 (13.6%)** | 5 (27.8%) | **1 (6.3%)** | **3 (20.0%)** |
| Kisumu West, Kenya | **27 (15.3%)** | **1 (1.7%)** | 0 (0.0%) | **0 (0.0%)** | **0 (0.0%)** |
| Mbeya, Tanzania | **57 (32.2%)** | **2 (3.4%)** | 4 (22.2%) | **5 (31.3%)** | **10 (66.7%)** |
| Abuja & Lagos Nigeria | **28 (15.8%)** | **24 (40.7%)** | 2 (11.1%) | **5 (31.3%)** | **2 (13.3%)** |
| Age (years) |  |  |  |  |  |
| 18-29 | 28 (15.8%) | 9 (15.3%) | **5 (27.8%)** | 2 (12.5%) | 2 (13.3%) |
| 30-39 | 62 (35.0%) | 17 (28.8%) | **2 (11.1%)** | 8 (50.0%) | 3 (20.0%) |
| 40-49 | 51 (28.8%) | 20 (33.9%) | **9 (50.0%)** | 2 (12.5%) | 7 (46.7%) |
| 50+ | 36 (20.3%) | 13 (22.0%) | **2 (11.1%)** | 4 (25.0%) | 3 (20.0%) |
| Sex |  |  |  |  |  |
| Male | 80 (45.2%) | 24 (40.7%) | 7 (38.9%) | 7 (43.8%) | 7 (46.7%) |
| Female | 97 (54.8%) | 35 (59.3%) | 11 (61.1%) | 9 (56.3%) | 8 (53.3%) |
| Currently employed |  |  |  |  |  |
| No | **93 (52.5%)** | **10 (16.9%)** | 7 (38.9%) | **5 (31.3%)** | 9 (60.0%) |
| Yes | **84 (47.5%)** | **49 (83.1%)** | 11 (61.1%) | **11 (68.8%)** | 6 (40.0%) |
| Education |  |  |  |  |  |
| Primary or less | **92 (52.0%)** | **25 (42.4%)** | 8 (44.4%) | 9 (56.3%) | 7 (46.7%) |
| Secondary or above | **85 (48.0%)** | **34 (57.6%)** | 10 (55.6%) | 7 (43.8%) | 8 (53.3%) |
| Enough food to eat in past 12 months |  |  |  |  |  |
| No | 46 (26.0%) | 16 (27.1%) | 6 (33.3%) | 4 (25.0%) | 2 (13.3%) |
| Yes | 130 (73.4%) | 43 (72.9%) | 11 (61.1%) | 11 (68.8%) | 13 (86.7%) |
| Missing | 1 (0.6%) | 0 (0.0%) | 1 (5.6%) | 1 (6.3%) | 0 (0.0%) |
| Distance from facility (km), median (IQR) | 8 (5-18.5) | 10 (5-20) | 5.5 (2-20) | 7.5 (3-15) | 8 (6.6-12) |
| Time since HIV diagnosis |  |  |  |  |  |
| <1 year | **0 (0.0%)** | 2 (3.4%) | **0 (0.0%)** | **1 (6.3%)** | 1 (6.7%) |
| 1 to 4 years | **98 (55.4%)** | 37 (62.7%) | **9 (50.0%)** | **8 (50.0%)** | 6 (40.0%) |
| 5 to 9 years | **52 (29.4%)** | 14 (23.7%) | **3 (16.7%)** | **2 (12.5%)** | 7 (46.7%) |
| 10+ | **27 (15.3%)** | 6 (10.2%) | **6 (33.3%)** | **5 (31.3%)** | 0 (0.0%) |
| Missing | **0 (0.0%)** | 0 (0.0%) | **0 (0.0%)** | **0 (0.0%)** | 1 (6.7%) |
| Duration on ART |  |  |  |  |  |
| ART naïve | **9 (5.1%)** | **6 (10.2%)** | **4 (22.2%)** | 2 (12.5%) | 3 (20.0%) |
| <6 months | **13 (7.3%)** | **5 (8.5%)** | **1 (5.6%)** | 0 (0.0%) | 0 (0.0%) |
| 6 months to <2 years | **52 (29.4%)** | **28 (47.5%)** | **3 (16.7%)** | 4 (25.0%) | 3 (20.0%) |
| 2 years to <4 years | **28 (15.8%)** | **3 (5.1%)** | **3 (16.7%)** | 5 (31.3%) | 1 (6.7%) |
| >4 years | **74 (41.8%)** | **17 (28.8%)** | **7 (38.9%)** | 5 (31.3%) | 7 (46.7%) |
| Missing | **1 (0.6%)** | **0 (0.0%)** | **0 (0.0%)** | 0 (0.0%) |  |

All data are presented as n (column percentage). P-values were calculated using Pearson’s chi-squared tests or Wilcoxon rank sum tests for continuous variables. Bold indicates significance at p<0.05.
